# Isolated Dorsal Dislocations of the Fourth and Fifth Carpometacarpal Joints: A Case Report and Review of Literature

**DOI:** 10.7759/cureus.12310

**Published:** 2020-12-26

**Authors:** Sreenivasulu Metikala, Paul Herickhoff

**Affiliations:** 1 Orthopaedics, Virginia Commonwealth University Health System, Richmond, USA; 2 Orthopaedic Surgery, Penn State Health Milton S. Hershey Medical Center, State College, USA

**Keywords:** carpometacarpal dislocation, closed reduction, percutaneous pinning

## Abstract

Dislocations of the carpometacarpal (CMC) joints without fracture are rare injuries. They commonly involve the fourth and fifth metacarpals of the dominant hand. Missed and incorrect diagnoses are quite frequent due to subtle clinical and radiological findings. Untreated cases may result in chronic disability due to long term pain and weakness in grip strength. Closed reduction is possible when performed early but can be unstable. We present a rare case of unstable dislocation of fourth and fifth carpometacarpal joints treated by closed reduction and percutaneous pinning.

## Introduction

Due to their unique anatomy and increased mobility, the fourth and fifth carpometacarpal (CMC) joints are more commonly injured than the radial three CMC joints. The usual mechanism of injury is direct blunt trauma to the ulnar side of the hand with a clenched fist such as punching a hard object or person. Fracture-dislocation of the CMC joint(s) occurs more frequently than dislocation without fracture, and dorsal dislocations are far more common than volar dislocations [1]. Diagnosis is often missed or delayed due to subtle findings on clinical and radiological evaluations [2,3]. Early recognition and prompt intervention are necessary to prevent impaired wrist and hand function. Computed tomography (CT) scan can be used to better interpret the injury pattern and also diagnose associated fractures [4,5]. Closed reduction is frequently successful if the patient presents early but can often be unstable, especially when more than one joint is involved. We present a rare case of unstable dorsal dislocation of the fourth and fifth CMC joints without fracture being managed by closed reduction and percutaneous Kirschner wire (K-wire) stabilization. 

## Case presentation

A 26-year-old male prisoner injured himself by punching a wall with his dominant right hand at his prison facility. He experienced immediate pain in the dorsal-ulnar wrist region and presented to the emergency department the next day. The patient denied previous injuries, numbness, or tingling of fingers. Clinical examination revealed tenderness of the dorsal-ulnar aspect of the right wrist with minimal swelling. No distal neurovascular deficits were found.

**Figure 1:**
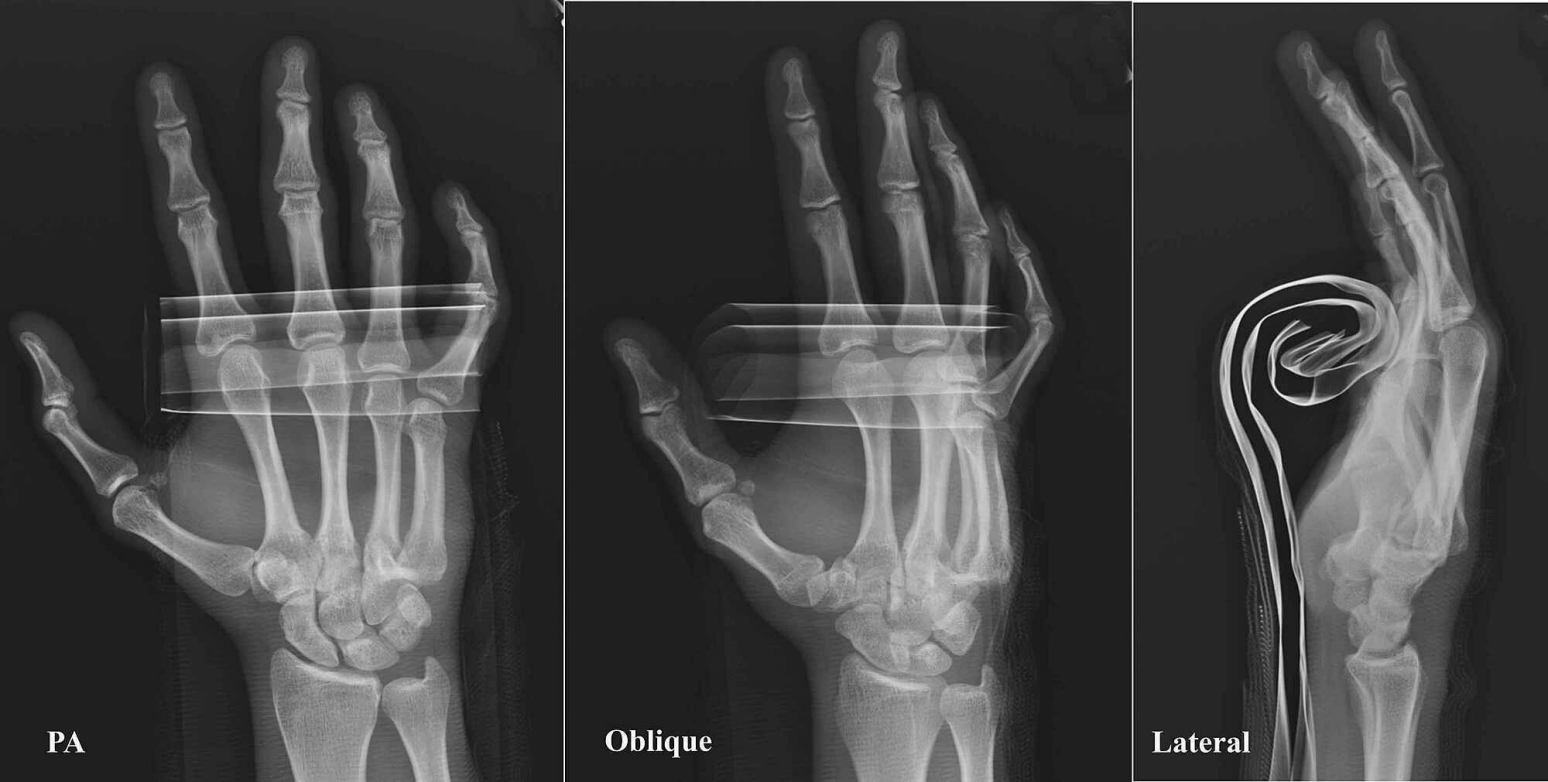
Posteroanterior (PA), oblique and lateral radiographic views with dorsal dislocation of 4,5 carpometacarpal joints

The posteroanterior, oblique, and lateral radiographs demonstrated dorsal displacement of the base of the fourth and fifth metacarpals at the corresponding CMC joints consistent with dislocation without fracture (Figure 1).

**Figure 2:**
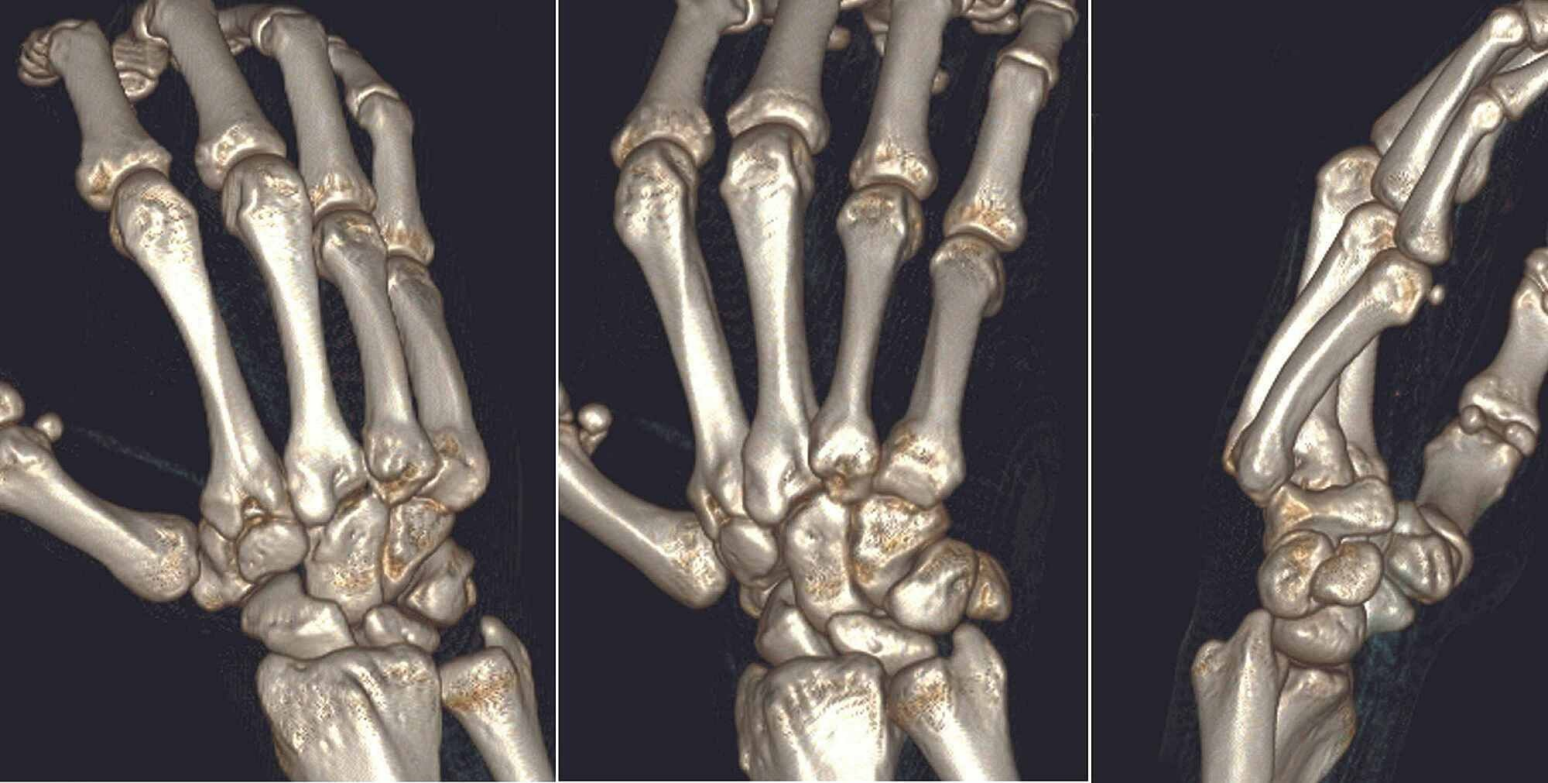
Three-dimensional CT reconstruction images confirming dorsal dislocations of 4,5 carpometacarpal joints with no associated fractures

A CT scan was performed that confirmed the above findings with no additional lesions (Figure 2). Closed manipulation attempted in the emergency department under light sedation anesthesia failed to achieve successful reduction. He was offered operative treatment, explaining the associated risks as well as benefits, and informed consent was obtained.

The patient was brought to the operating room four days post-injury. He was positioned supine and the right hand was placed on a hand table. Under general anaesthesia, a closed reduction was attempted using longitudinal traction with direct thumb pressure, and the displaced bones were reduced. The fluoroscopic views confirmed the relocation of the fourth and fifth CMC joints but were found to translate dorsally with wrist flexion. In light of instability, a decision was made to perform pin fixation. Under fluoroscopic guidance, a 0.062-inch Kirschner wire (K-wire) was inserted through a stab incision on the dorsal aspect of the base of the fourth metacarpal and was driven across the reduced CMC joint into the body of the hamate.

**Figure 3:**
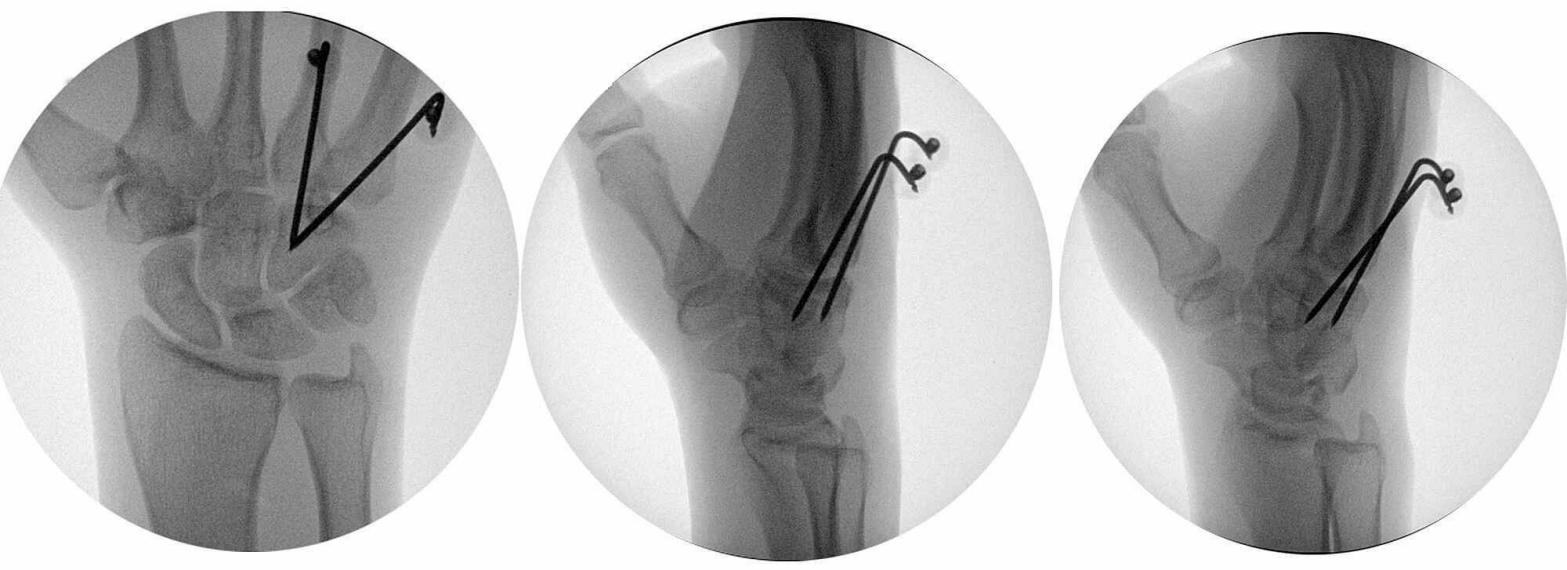
Intraoperative fluoroscopic images confirming well-reduced 4,5 carpometacarpal joints being stabilized by two K-wires

A second K-wire was inserted, in a similar fashion, stabilizing the fifth CMC joint to the body of the hamate (Figure 3). The stability of reduction was confirmed in dynamic views and the trailing ends of the K-wires were protected with Jürgen pinballs. A well-padded short arm plaster cast was applied to keep the hand in the intrinsic-plus position and the patient was discharged back to the prison facility on the same day.

**Figure 4:**
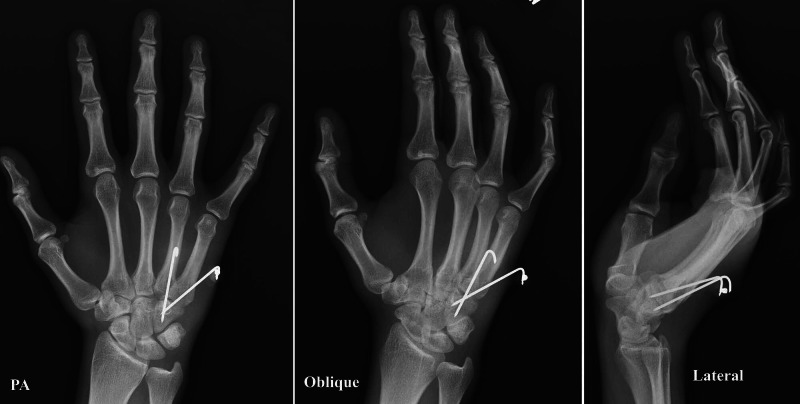
Postoperative posteroanterior (PA), oblique and lateral follow-up radiographs at 3.5 weeks shortly before K-wires removal showing maintenance of reduction

The plaster and K-wires were removed in the office at 3.5 weeks postop after repeating the radiographs (Figure 4). Superficial infection was noted at the pin insertion sites and the patient was advised a one-week course of oral antibiotics along with local wound care. His hand was positioned in a removable splint and range of motion exercises of the wrist and fingers were begun. He was examined at the six-week mark with a resolved infection.

**Figure 5:**
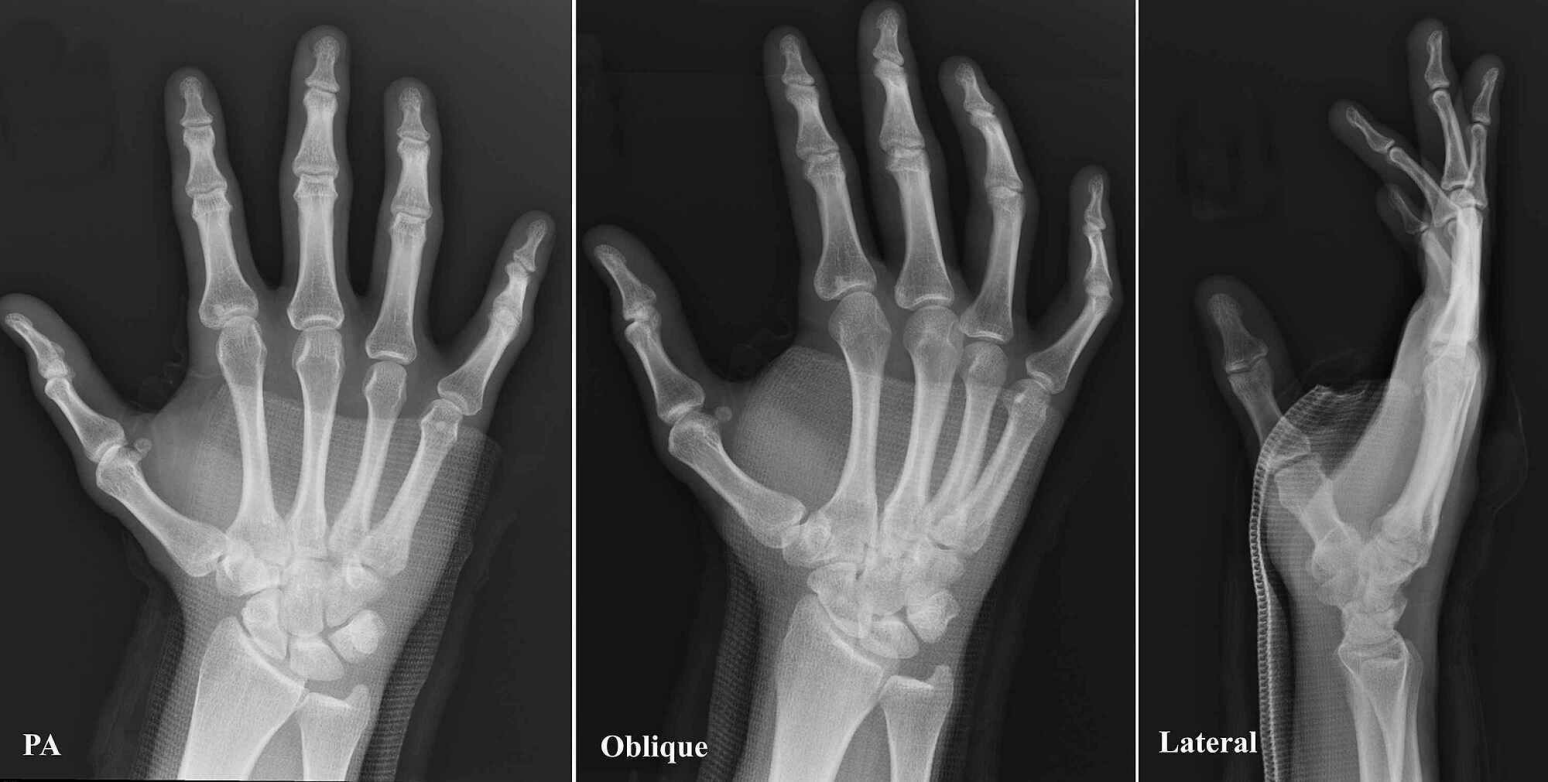
Postoperative posteroanterior (PA), oblique and lateral follow-up radiographs at 6 weeks showing anatomical position of 4,5 carpometacarpal joints

The posteroanterior, oblique, and lateral radiographs had confirmed the anatomical position of 4-5 CMC joints (Figure 5). He also had regained full range of wrist motion and grip strength. Further follow-up was not possible due to the onset of the coronavirus disease 2019 (COVID-19) pandemic and the release of the patient from prison with no contact information.

## Discussion

Concurrent dislocation of four and five CMC joints is an uncommon injury and careful attention must be paid to clinical and radiological findings if the injury is not to be missed. The history of a clenched fist striking a hard surface on the ulnar border, like in this patient, can be a clue to the diagnosis. The posteroanterior radiograph reveals loss of parallelism between the CMC joints with overlapping articular surfaces [1]. The true lateral view is critical, especially to identify subtle CMC joint subluxations, and must be carefully evaluated through the overlapping of bony structures [6]. The oblique view is often helpful to better evaluate the ulnar CMC joints [7]. However, the particulars of the degree of joint subluxation and fracture pattern are often difficult to understand solely by radiographs. Ultrasound can be a relatively inexpensive, and quick investigation providing dynamic examination without radiation, but is operator-dependent with poor repeatability [8]. Thus, a CT scan is the best investigation that confirms the diagnosis and reliably demonstrates the joint surfaces and associated fracture patterns [5].

Closed reduction can be successful when attempted within seven to 10 days of the injury. An open reduction is frequently necessary for delayed presentations and fracture-dislocations due to massive edema, interposed fracture fragments, and/or ligamentous structures [9]. An unstable CMC joint dislocation usually requires K-wire stabilization. Few cases of closed reduction and immobilization in a well-molded plaster cast without internal fixation were described in the literature [10,11]. However, loss of reduction with secondary dislocation or partial subluxation is possible within the first two weeks following manipulation [11]. For that reason, frequent postoperative monitoring with serial radiographs is essential, which may not be possible in prison inmates due to the challenges in bringing them to the office every week. Further, interpretation of the radiographs can often be difficult as they are usually done through the plaster. Intraoperatively, a precise K-wire insertion technique is important for achieving stability of the reduction and avoiding complications. The single-pass approach should be executed with appropriate planning since multiple attempts of pinning could jeopardize the stability and/or create additional iatrogenic comminution. According to an anatomical study by Bhandari et al., the average dorsal angles of the fourth and fifth metacarpals are 11 degrees and 12 degrees respectively [12]. The pin driver, accordingly, should be gradually brought close to the metacarpal as the K-wire advances. Pin tract infection, as developed in this case, may complicate the postoperative course even with the meticulous surgical technique. A recent study reported an overall 7% incidence of K-wire pin site infection in hand and wrist fractures that required additional treatment with antibiotics or early K-wire removal or reoperation [13]. Poor hygiene in the prison environment might have predisposed to this infection.

## Conclusions

A high index of suspicion is necessary when a patient presents with a history of punching a hard object resulting in ulnar-sided wrist pain. Careful evaluation of radiographs reveals the diagnosis and a CT scan can be a useful adjunct to better interpret the joint surfaces. It is possible to achieve a closed reduction in an early presentation but the stability of the reduction has to be determined. K-wire stabilization becomes necessary if there is any suspicion of instability. Meticulous surgical technique and attentive post-operative care can result in a successful outcome.
